# TP53-PTEN-NF1 depletion in human brain organoids produces a glioma phenotype *in vitro*

**DOI:** 10.3389/fonc.2023.1279806

**Published:** 2023-10-10

**Authors:** Sanjay K. Singh, Yan Wang, Ahmed Habib, Mamindla Priyadarshini, Chowdari V. Kodavali, Apeng Chen, Wencai Ma, Jing Wang, N. U. Farrukh Hameed, Baoli Hu, Gregory N. Fuller, Scott M. Kulich, Nduka Amankulor, Rivka R. Colen, Lincoln A. Edwards, Pascal O. Zinn

**Affiliations:** ^1^ Department of Neurosurgery, MD Anderson Cancer Center, Houston, TX, United States; ^2^ Department of Neurosurgery, University of Pittsburgh Medical Center, Pittsburgh, PA, United States; ^3^ Department of Radiology, University of Pittsburgh Medical Center, Pittsburgh, PA, United States; ^4^ Department of Bioinformatics, MD Anderson Cancer Center, Houston, TX, United States; ^5^ Department of Pathology, MD Anderson Cancer Center, Houston, TX, United States; ^6^ Department of Pathology, University of Pittsburgh Medical Center, Pittsburgh, PA, United States; ^7^ Department of Neurosurgery, Hospital of the University of Pennsylvania, Philadelphia, PA, United States

**Keywords:** organoid, nestin, glioblastoma, TP53, PTEN, and NF1 tumor suppressors, proneural GBM subtype

## Abstract

Glioblastoma (GBM) is fatal and the study of therapeutic resistance, disease progression, and drug discovery in GBM or glioma stem cells is often hindered by limited resources. This limitation slows down progress in both drug discovery and patient survival. Here we present a genetically engineered human cerebral organoid model with a cancer-like phenotype that could provide a basis for GBM-like models. Specifically, we engineered a doxycycline-inducible vector encoding shRNAs enabling depletion of the TP53, PTEN, and NF1 tumor suppressors in human cerebral organoids. Designated as inducible short hairpin-TP53-PTEN-NF1 (ish-TPN), doxycycline treatment resulted in human cancer-like cerebral organoids that effaced the entire organoid cytoarchitecture, while uninduced ish-TPN cerebral organoids recapitulated the normal cytoarchitecture of the brain. Transcriptomic analysis revealed a proneural GBM subtype. This proof-of-concept study offers a valuable resource for directly investigating the emergence and progression of gliomas within the context of specific genetic alterations in normal cerebral organoids.

## 1 Introduction

Glioblastoma (GBM) remains the most fatal primary brain tumor in adults, with a median survival of 2 years despite maximum surgical and medical management (1, 2). GBM patient tumors and the derived glioma stem cells are a restricted and limited resource, thereby slowing progress in addressing concerns of resistance to current therapy, disease progression, and drug discovery ultimately affecting patient survivorship (3).

The immense cell biological and genetic complexity of GBM has underscored the need for tractable pre-clinical models that are readily engineered with signature genetic alterations that recapitulate the cytoarchitectural features and malignant evolution of human GBM. Such model systems, coupled with existing platforms, would advance our understanding of disease pathogenesis, accelerate drug discovery, and improve outcomes for GBM patients.

Accurate modeling of human GBM should encompass human cancer stem cells and GBM subtype classification and should maintain the species-specific aspects of the tumor microenvironment in order to explore therapeutic resistance, disease progression, and targeted vulnerabilities. Various human preclinical model systems have been developed that have played an important role in advancing our understanding of GBM and have served as the backbone for screening candidate therapeutic targets. For example, pa-tient-derived xenografts (PDX) in mice fulfill some features of human GBM, such as tumor infiltration, although this model is limited by cross-species differences in the tumor microenvironment (4). Additionally, 2D cultures of human tumor cells have provided a system for the study of glioma stem cells (5–8) yet lack the host-cancer cell interactions present in the brain parenchyma (9, 10).

More recently, 3D human cerebral organoid cultures have emerged as a platform for modeling GBM including related pathological processes (11, 12). Human cerebral or-ganoid cultures represent a model technology that can be generated from human embryonic stem cells (hESCs), or induced pluripotent stem cells, allowing investigators to address multiple questions concerning human brain development and disease, including GBM (13, 14). We and others have played a role in contributing to such models (15–17). The 3D GBM cerebral organoid models fulfill the criteria of possessing cancer stem cells, mirroring the GBM subtype, and providing a species-specific tissue context in which to study interactions across various normal cell types and cancer cells. Such attributes obtained within 3D organoid models are similar to genetically engineered mouse models (GEMMs), which enable tumor microenvironment interactions in the same species (18–21). GEMMs have established the genetic tractability of such systems, as well as providing the ability to observe the tumor initiation process at the microscopic level although the human component of such a system is lacking (18, 19, 22).

In this study, we present a genetically engineered human cerebral organoid with a cancer-like phenotype that could provide a basis for GBM study. we report the engineering of a doxycycline (Dox) inducible TP53-PTEN-NF1 multiplex shRNA construct (ish-TPN) in a defined, Nestin-positive progenitor cellular niche within the human brain organoid. The three tumor suppressor genes were identified as significantly mutated genes in a comprehensive characterization of more than 500 glioblastoma tumors (GBMs) and the key feature of the design is an inducible and genetically defined brain cancer organoid that permits the direct observation of human tumor initiation in future studies (23).

## 2 Materials and methods

### 2.1 Cell lines

H1 & H9 human embryonic stem cell line was purchased from WiCell Research Institute Inc (Madison, Wisconsin, USA). The human embryonic stem cell line was maintained in 6-well plates coated with Matrigel with the supplemented mTeSR2 medium (Stem Cell Technologies).

### 2.2 Lentiviral transduction of hESC and FACS

The lentiviral particles were packaged using plasmids in the Tet-On 3G inducible kit from Takara. Tet-on plasmid promoter was replaced with the nestin promoter. ShRNA sequences in the target vector can be found in **Supplemental Table 1**. hESCs were plated on 24 well plates and then allowed to adhere overnight. 2µl of the virus solution (1 x 106 viral particle/µl) containing both the vectors was added to each well and the cells were incubated for 24 hrs then media was changed to a fresh virus-free medium. The cells were incubated for an additional 5 days and then selected for puromycin selection (0.5ug/mL). After puromycin selection, the hESCs were sorted for GFP then expanded and cryopreserved.

### 2.3 Organoid culture

Cerebral organoids were generated from human embryonic stem cells (hESC) using a modified protocol (13, 16). To summarize; our protocol is divided into 3 phases. Phase 1. Was generating embryoid bodies from transduced hESCs and germ layer differentiation (days0-5) using mTeSR1 medium (STEMCELL Cat#85850) then phase 2 was neural induction phases using neural induction media (STEMCELL Cat#05835) to induce the neural ectoderm within the organoids (days 6-10) then we transfer the organoid into differentiation media (16) to start phase 3 which is the differentiation phase.

### 2.4 Immunofluorescence

Immunostaining was performed using standard procedures for tissue cryosections. The sections were blocked in blocking buffer (5% Normal Goat Serum/3% BSA/PBS/0.01% Tween-20), then incubated in primary antibody solution in blocking buffer at 4°C overnight. After washing in PBS-T (0.1% Tween-20), sections were incubated with secondary antibody solution for 2hr and washed with PBS-T, then mounted with ProLong™ Gold Antifade Mounting (Thermo Fisher). A total of 60 organoids were included in this analysis (TP53 n=15), (PTEN n=16), (NF1 n=20), (Ki-67 n=3), (Nestin n=3), and (GFAP n=3) after applying the universal background correction for 200x200μm Region of Interest (ROI) grid and a universal threshold. The segmentation of the organoid section into a grid resulted in equal regions of interest (ROIs), ranging from 384 to 480 ROIs for each of the four test groups per gene: control Dox-, control Dox+, ish-TPN Dox-, and ish-TPN Dox+. The resulting data from these ROIs were plotted using standard error of mean bar graphs.

### 2.5 qRT-PCR

We used SYBR qRT-PCR to estimate gene expression for TP53, PTEN, NF1, Nestin, and Ki-67, (for primer sequences used, see **Supplementary Table 2**). The experiment was performed according to the manufacturer’s instructions.

### 2.6 Microscopy

All images were captured using the Nikon Eclipse Ti2-E inverted microscope system (25mm field of view (FOV), and image analyses were performed with the NIS-Elements AR (Advanced Research) software; both were purchased in 2019.

### 2.7 RNA-seq analysis

RNA was prepared using TRIzol from Dox-treated and Dox-untreated organoids. Total RNA from organoids was purified by Direct-zol™ RNA MicroPrep (Zymo Research), and cDNA libraries were prepared using the TruSeq RNA Sample Prep kit (Il-lumina). RNA sequencing was performed using a HiSeq 2500 Sequencing System (Illumina). Sequenced reads were quality-tested using FASTQC, and STAR was used to align the RNA seq data to the human genome data hg38, generating bam files. Using Samtools, bam files were further converted to Sam files. These were then used with the HTSeq software to count the number of reads and to generate the raw gene expression matrix data. The data were normalized using variance stabilizing transformation in the DESeq2 R package. We used a box plot, hierarchical clustering, and PCA (Principal Component Analysis) plot to view the data for the purpose of quality control. Significant differences between the means of each gene’s expression for Dox-negative and Dox-positive groups were determined using the t-test. The differentially expressed genes were selected based on both the t-test p-value (threshold 0.05) and Benjamini-Hochberg false discovery rate (BH-FDR) value (0.1). For total RNA-Seq data, functional analysis of differentially-expressed genes (DEG) was performed using DESeq2 analysis was carried out on the gene sets enriched in Dox-negative and Dox-positive groups using Gene Set Enrichment Analysis (GSEA) ver. 2.0.

### 2.8 Copy Number Alteration (CNA) calling analysis

CNA calling on RNA-Seq data was performed using the SuperFreq algorithm. The SuperFreq pipeline is used for cancer sequencing analysis and performs clonality tracking by integrating the identification of somatic single nucleotide variants (SNVs) and copy number alterations (CNAs) (Flensburg et al., 2020). The algorithm works with or without a matched normal and can simultaneously detect and track somatic mutations in multiple samples from the same sample, resolving overlapping CNA calls by cross-checking variant calls and separating somatic from germline variants. RNA-Seq samples in our analysis were aligned using the STAR aligner with default parameters as the settings and the hg19 genome as the reference genome for alignment. Our final RNA-Seq dataset after quality control passing (QC > 75%) consisted of 17 samples, of which ish-TPN Dox+ (n=9), Control Dox (n=3), and ish-TPN Dox- (n=5). These samples were used for CNA calling using SuperFreq. SuperFreq version 1.4.0 (available at https://github.com/ChristofferFlensburg/SuperFreq) was used, and the analysis was carried out in R statistical programming language (version 3.6.0).

### 2.9 Statistical analysis

Data are presented as mean ± SEM and were analyzed using paired two-tailed Student’s t-test to determine significance. For statistical significance, p values < 0.05 were considered significant. Graphs were made using GraphPad Prism V.8.0.

## 3 Results

### 3.1 Generation and characterization of cerebral organoids from human embryonic stem cells

**Figure 1:**
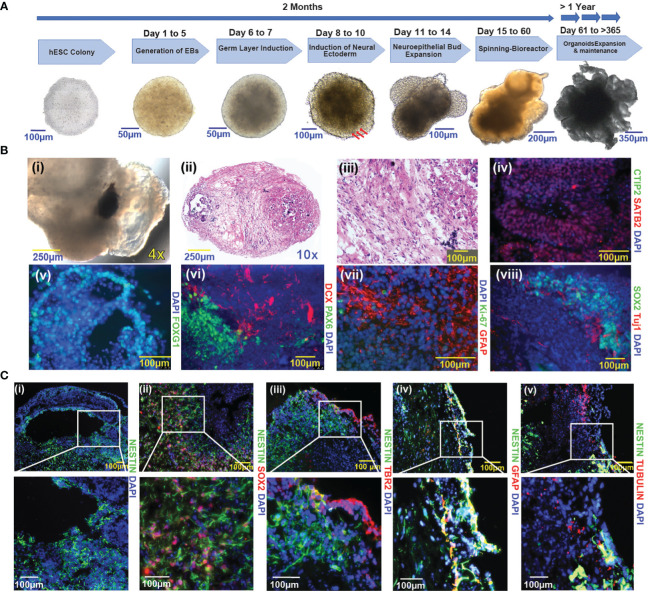
Generation and characterization of COs from hESCs **(A)** Schematic representation of the cerebral organoid generation protocol and timeline. A representative image of an undifferentiated hESC colony is shown in the first panel to the left. Sequential images are representative of the key steps in the course of cerebral organoid formation. Three red arrows indicate the induction of neural ectoderm. **(B)** Characterization of cerebral organoids. (i-viii): (i): Pig-mented retinal epithelium tissue; (ii-iii): H&E-stained sections of cerebral organoid (10x and 20X magnification respectively); (iv): CTIP2, SATB2; (v): FOXG1; (vi): PAX6 and DCX (vii): Ki-67 and GFAP; (viii): SOX2 and TUJ1. **(C)** Immunofluorescence images of cerebral organoids represent the distribution of Nestin-positive cells vs other pluripotency factors (i-v), (i): Nestin; (ii): Nestin and SOX2; (iii): Nestin and TBR2; (iv): Nestin and GFAP; (v): Nestin and Tubulin. Images were taken with a Nikon Eclipse Ti2-E microscope; nuclei are highlighted by DAPI staining in figures (**B**iv–**C**v). The magnification given in the caption reflects size before reduction.

Using the well-established protocol of Lancaster and colleagues with modifications, we generated a panel of human embryonic stem cell (hESC)-based cerebral organoids (13) (**Figure 1A**). These hESCs used for cerebral organoids differentiation were maintained in self-renewing conditions (see methods section) and confirmed to express pluripotency factors Nanog, Sox2, and Oct4 (**Supplementary Figure 1A**). Consistent with multi-potency, cerebral organoids (COs) generated from hESCs contain multiple neural cell lineages. We observed pigmented retinal epithelial tissue during embryoid formation in many COs (120 out of 384) (**Figure 1A**i). Histological analysis shows evidence of regional heterogeneity within the COs (**Figures 1B**ii, iii). SATB2, a marker of late post-natal superficial neuronal expression is localized to specific cell layers (**Figure 1B**iv), which is distinct from areas containing cells positive for CTIP2 (**Figure 1B**iv), a marker of early post-natal neurons. FOXG1 (**Figure 1B**v) is expressed in areas with morphology reminiscent of the primary brain vesicle during embryonic development. Cells positive for DCX (**Figure 1B**vi), a marker of newborn neurons, are organized in areas distinct from PAX6-positive cells (**Figure 1B**vi), consistent with prosencephalic differentiation. GFAP (**Figure 1B**vii), a marker of differentiated astrocytes, was expressed in COs, while the proliferative marker Ki-67 (**Figure 1B**vii) was largely negative. The SOX2-positive Ki-67-negative status suggested a quiescent cell state (**Supplementary Figure 8**). In the more developed COs, SOX2-positive neural stem cells (NSCs) were retained and were located in the vicinity of TUJ1-positive neuronal cells (**Figure 1B**). Notably, cells expressing neural stem cell markers localized to regions juxtaposed to fluid-filled cavities, a pattern that to some extent reminiscent of the neural stem cell niche in the subventricular zone (SVZ) of the human brain (**Figure 1C**i) (6, 24). GFAP expression was not readily observed in cells expressing Nestin, an early neuronal marker. Radial glial and SVZ markers were captured by Tubulin, NESTIN, and TBR2 (**Figure 1C**). The cultured COs were not discernably hypoxic, as evidenced by the lack of hypoxia markers HIF1-alpha and CAIX (data not shown).

### 3.2 Niche-specific targeting and transgene expression

**Figure 2:**
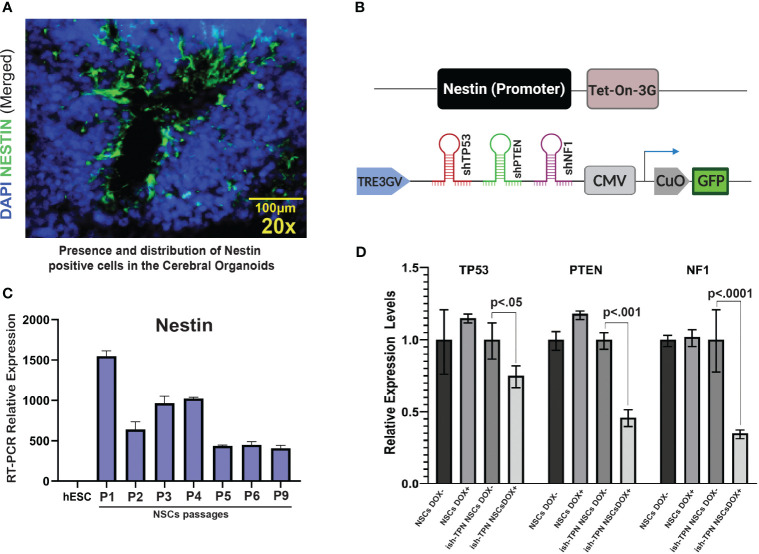
Validating the cell type-specific inducible multiplex shRNA driven system **(A)** Immuno-fluorescence image of a cerebral organoid within the SVZ-like region at 20x magnification showing Nestin-expressing cells (green) and nuclei stained with DAPI (blue). **(B)** Illustration of inducible and cell type-specific lentiviral multiplex shRNA construct designed to target specific genes. (Top) Expression of Tet-On-3G-IRES cassette (Nestin promoter). (Bottom) Multiplex shRNAs targeting TP53, PTEN, and NF1 under the TRE3GV promoter. Constitutive expression of GFP to allow for enrichment of transduced cells. **(C)** Passages of hESCs-derived neural stem cells express Nestin. qRT-PCR results show the relative abundance of Nestin expression in hESCs-derived NSCs over 9 passages as compared to parental hESCs **(D)** qRT-PCR demonstrating knockdown of TP53, PTEN, and NF1 transcripts in neural stem cells after infection with shRNA constructs targeting TP53, PTEN, and NF1. n=3 for each condition; p values are as indicated. The magnification given in the caption reflects size before reduction.

We sought to assess the feasibility of directing transgene expression to specific cell types: Nestin promoter was used to drive transgene expression to Nestin-positive cells residing within the SVZ-like areas of the human CO model (**Figure 2A**). The Nestin promoter was selected based on its expression in NSCs (25, 26), which are present within the SVZ-like region of the brain (27, 28) and are a known anatomic site where GBM can originate (5, 6, 29–31). Compared to the hESCs, differentiated neural stem cells demonstrated a 1000-fold increase in Nestin expression, and the expression was maintained at high levels on subsequent passages (**Figure 2C**). We constructed two lentiviral plasmids: (i) Tet-On-3G-IRES cassette under the control of the human Nestin promoter, and (ii) Dox-inducible TRE3GV promoter directing expression of three tandem shRNAs targeting TP53, PTEN, and NF1 (**Supplementary Table 1**). The latter plasmid also contained a constitutively-expressed GFP gene to enable the selection of transduced hESCs (**Figure 2B**). Following lentiviral production and trans-duction of hESCs, cell sorting captured hESCs with 30% highest levels of GFP (approximately 4500 GFP-expressing hESCs). These hESCs containing the constructs or corresponding controls were differentiated into NSCs first, treated with doxycycline (NSC Dox+) to validate the functionality of the inducible expression system, We used qRT-PCR (**Figure 2D** and **Supplementary Table 2**) to test the knockdown efficiency of the targets gene after turn on the nestin promoter driven shRNAs expression. We also confirmed the protein expression knockdown by Western blot analysis (**Figure 2E**).depletion of TP53, PTEN, and NF1 only happens in the Dox+ but not in the Dox- NSCs. In addition, ish-TPN Dox+ NSC cultures exhibited a higher proliferation (as determined by Ki-67 staining) and Nestin expression in comparison to ish-TPN Dox-NSC cultures. (**Supplementary Figure 2A**, p=0.0436 and **Supplementary Figure 2B**, p=0.0419).

### 3.3 Neural progenitor cell targeting in human cerebral organoids produces GBM-like phenotype

**Figure 3:**
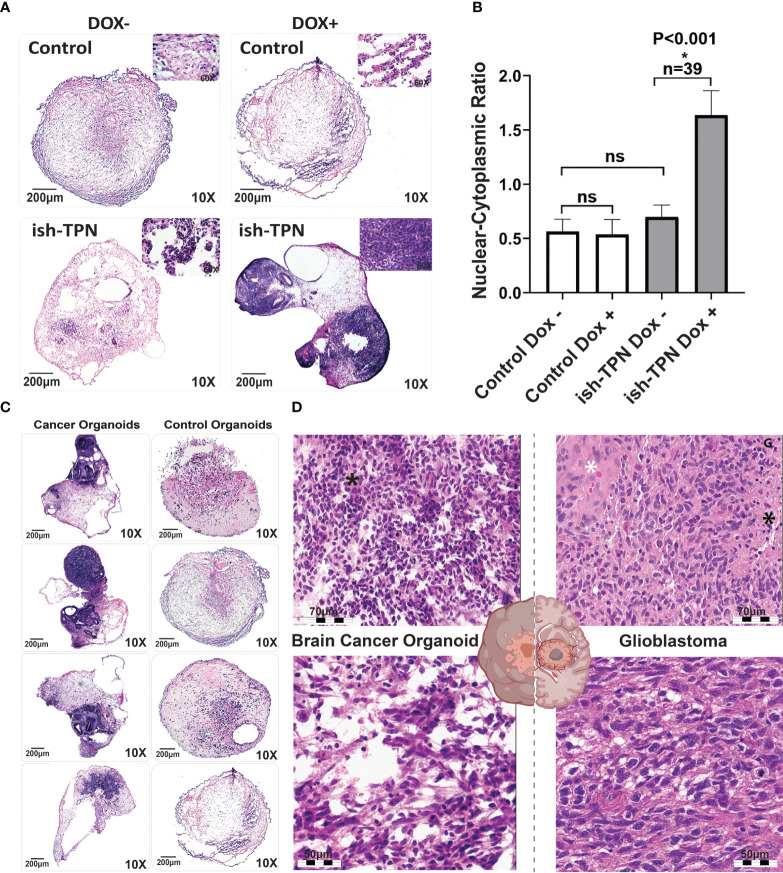
Organoid cancer model characterization **(A)** H&E-stained control (no ish-TPN), and ish-TPN containing 2-month-old COs before (left) and (right) after exposure to doxycycline (1ug/mL medium) for 2 weeks. **(B)** Quantification of the nuclear-to-cytoplasmic ratio of the control (no ish-TPN) vs ish-TPN containing doxycycline-induced cancer organoids with and without doxycycline treatment. Data are shown with error bars as ± SEM, n=39, ns=non-significant. **(C)** H&E-stained representative sections of control and ish-TPN containing doxycycline-induced cancer organoids (10x magnification). **(D)** H&E-stained sections of ish-TPN containing doxycycline-induced cancer organoids (left) vs human glioblastoma (right; white asterisk=endothelial proliferation; black asterisk=necrosis). The magnification given in the caption reflects size before reduction. ns, not significant.

We opted to exploit targets that relied upon our experience with GBM and within genetically-defined COs (32). To assess if the targeted knockdown of tumor suppressor genes would generate glioblastoma-like histology *in vitro*, we targeted the following tumor suppressor genes, which have been identified as driver mutations in patient tumors: TP53, PTEN, and NF1 (33, 34). We transduced hESCs with the Dox-inducible ish-TPN expressed under a Nestin promoter to generate COs. Mature COs (> 2 months old) with ish-TPN were induced with Dox or left uninduced for two weeks. Mature organoids were then processed for morphological characterization (**Figure 3**). H&E-stained sections demonstrated that all organoids consisted of an admixture of neuroepithelial structures, relatively solid hypercellular areas, and a background of the relatively hypocellular stroma. Morphologic assessment of these mature COs demonstrated striking Dox-induction-dependent morphologic features in mature COs transduced with ish-TPN. Quantification of the nuclear-to-cytoplasmic ratio showed significant differences only with the Dox-induced ish-TPN mature COs (p <.001, **Figure 3B**). As shown in (**Figure 3C**), mature COs targeted with ish-TPN and treated with Dox demonstrated increased numbers of hypercellular foci (**Figure 3C**), with the neuroepithelial structures exhibiting increased architectural complexity and frequently containing luminal apoptotic-type debris cuffed by hypercellular stroma. Induced mature organoids exhibited apoptotic-type debris in hypercellular foci more frequently than uninduced mature organoids. Induced organoids also demonstrated increased cell density within the hypercellular foci (3.8 fo-ci/organoid vs. 0.7 foci/organoid) (**Figure 3C**). The morphologic appearance was reminiscent of glioblastoma (**Figure 3D**) with respect to hypercellularity and infiltration into the less cellular areas of the organoid. We also observed multinucleated GBM cells. Clinical neuropathologists were blinded as to the nature of the ish-TPN-containing mature COs that underwent Dox induction (cancer organoids) and correctly categorized the organoids as either cancer organoids or control organoids (not ish-TPN containing) over 80% of the time.

### 3.4 Inferred mutational landscape of human GBM-like cerebral organoids

**Figure 4:**
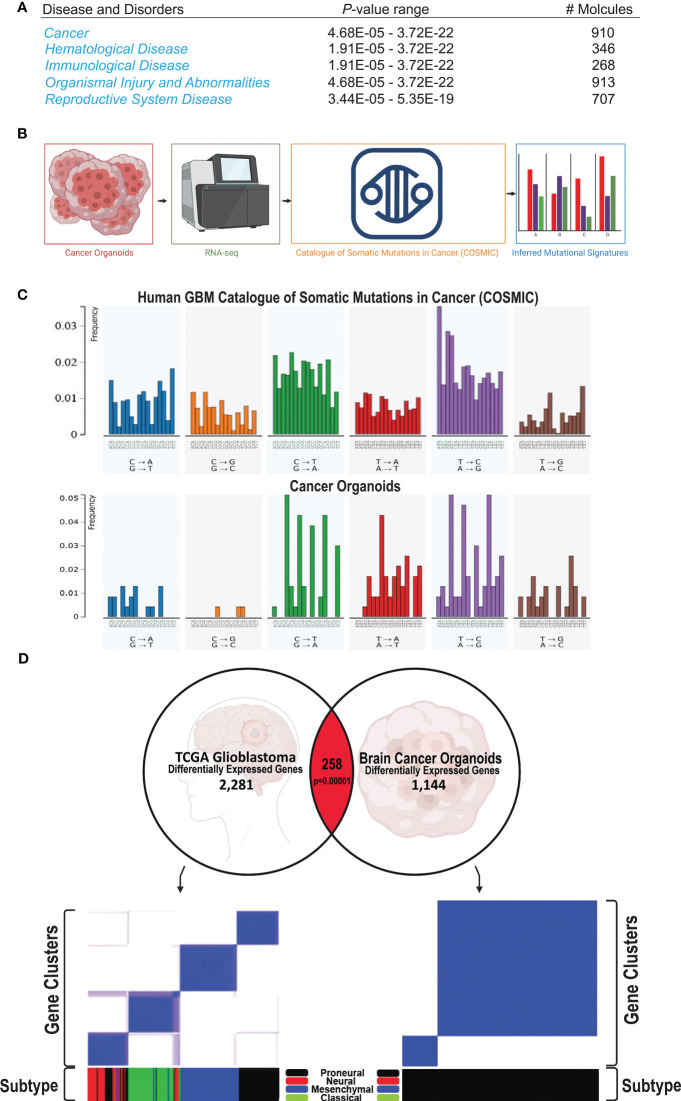
Human GBM COs signature and subtype **(A)** Ingenuity Pathway Analysis from RNA-seq of 17 COs shows that Cancer is the primary phenotype, being highly significant as shown by the p-value range. **(B)** Schematic of the process of subjecting the human COs to RNA-seq analysis; the resulting mutational output is compared against mutational signatures of the Catalogue of Somatic Mutations in Cancer (COSMIC); the mutational signature from our human COs can be subsequently visualized. **(C)** Representative comparison of the somatic mutational signature of our human GBM-like CO (top) compared to the COSMIC mutational signature (bottom). **(D)** Correlation of significantly associated genes in our cancer model with the GBM TCGA gene expression database. 154 genes are altered in the same direction as in the GBM TCGA gene expression profile out of 258 matched genes (p=0.00001).

The transcriptomes of human GBM-like COs and controls were profiled using RNA sequencing (RNA-seq) to identify differentially-expressed RNAs, inferring copy number and somatic mutational landscapes. Across all 9 human GBM-like COs, a total of 2,513 somatic mutations were identified. These mutations were mainly comprised of sin-gle-nucleotide variants (SNVs), including 1,904 missense mutations, 316 splice site mutations, 272 insertions, 10 deletions, and 11 mutations were not present in the 3 control organoids treated with Dox or the 5 ish-TPN COs that were Dox-. A total of 21 genes were identified as having a significant (p <.01) frequency of mutation, amplification, or deletion (**Supplementary Figure 3A**). PDGFRA, APOA2, ITLN1, FBN1, TTN, CDKN1A, PIK3CG, ITGB2, C10orf90, and GAD2 were among the most notable altered genes. These genes and their corresponding gene products comprised growth factor receptors, extracellular matrix proteins, cell cycle regulators, and cell signaling cascade effectors involved in cell proliferation and survival. In addition, Ingenuity Pathway Analysis revealed that cancer was the main phenotype within our human GBM-like COs (**Figure 4A**). From our RNA-seq analysis of human GBM-like COs, we quantified somatic mutational profiles (**Figure 4B**). We initially determined the 6 base pair substitution spectrums for GBM-like COs and determined the relative contribution of each of the 6 base substitution types over all samples (**Supplementary Figure 3B**). Prominent mutations were observed in T>C transitions, making up the bulk of mutations at 43%, followed by C>T transversions, 24% overall, but 6% of these mutations were specifically for C>T at CpG sites (**Supplementary Figure 3B**). We then determined the mutational signatures from all 96 trinucleotide changes across human GBM-like COs. The mutational signature was consistent with the SBS5 mutational signature in the Catalogue of Somatic Mutations in Cancer (COSMIC), which contains GBM as a disease entity (**Figure 4C**). Comparison of the mutational profile of human GBM-like COs to that of SBS5 indicated a cosine similarity of 0.92 (**Supplementary Figure 3C**, bottom).

RNA-seq data revealed that human GBM-like COs fell into 3 categories representing 3 different conditions. Group 1 represented the ish-TPN Dox+ human GBM-like COs (n=9); Group 2 represented the control Dox+(no ish-TPN) human COs (n=3), and Group 3 represented the ish-TPN Dox- COs (n=5). Group 2 control Dox-treated COs were distinct from Group 1 ish-TPN Dox-treated human GBM-like COs, as expected, while group 3 no Dox ish-TPN-containing COs represented a position between groups 1 and 2 as judged by principal component analysis (PCA) of all of the transcriptional data of each human CO (**Supplementary Figure 4A**). To validate our PCA results, we examined the inter-and intra-group variability using Pearson’s correlation coefficient (**Supplementary Figure 4B**) (35, 36). Similar to our PCA result, we found a distinct sample and gene clustering of COs containing ish-TPN with and without Dox treatment and control Dox COs without ish-TPN constructs with Dox treatment (**Supplementary Figure 5A**). We next determined differentially expressed genes in our human GBM-like COs treated with doxycycline. The resulting volcano plot showed that our human GBM-like COs had several upregulated (red) and downregulated (blue) genes (**Supplementary Figure 5B**). Genes with an absolute log-fold change greater than >0.4, comprising the top 133 genes, were used for further analysis.

In order to begin to understand the nature and the pathways involved in the formation and progression of our human GBM-like organoid cancer, we sought to determine the GBM subtypes present in our model system (37, 38). We analyzed the differential-ly-expressed genes from our human GBM-like CO model and compared these genes to the GBM TCGA gene expression dataset. 258 genes that matched GBM, of which 154 genes were altered in the same direction (**Figure 4D**, top, p=0.0001). We then confirmed that the GBM TCGA data could be placed into the expression signatures of the proneural, neural, classical, and mesenchymal GBM subtype classification system (37) (**Figure 4D**, bottom left). We subsequently performed the same analysis on our data set to determine the GBM subtype classification of our human GBM-like organoids. This classification analysis revealed that our human GBM-like organoids had a proneural expression sig-nature (**Figure 4D**, bottom right, **Supplementary Figures 5C, D**). This is consistent with both our initial gene set enrichment analysis (**Supplementary Figure 5E**) and previously published work on GBM Cos (32, 39, 40). We found loss of the TP53 gene within the proneural subtype, as expected given that we targeted this gene. PTEN was lost, as was NF1, in the proneural subtype (37, 38). In addition, a hallmark feature of this subtype is the amplification of PDGFRA; the amplification was modest in our human GBM-like COs. CDKN1A, which is also a feature of the proneural subtype was found in our GBM-like COs (**Supplementary Figure 3A**). Another hallmark feature that has been described for the proneural GBM subtype is IDH1 point mutations. We did not appreciate any IDH1 mutations; however, IDH1 point mutations are not typically present if PDGFRA alterations are also present (37, 41). Gene ontology (**Supplementary Figure 6A**) also revealed similarities to the gene ontology reported for the proneural GBM subtype, including cell cycle and developmental regulation (37, 41). Differentially expressed genes showed substantial enrichment for disease ontology pathways involving cancer, including GBM (**Supplementary Figure 6B**).

### 3.5 Lineage-specific ish-TPN activation in human GBM-like organoids and pathway effects

**Figure 5:**
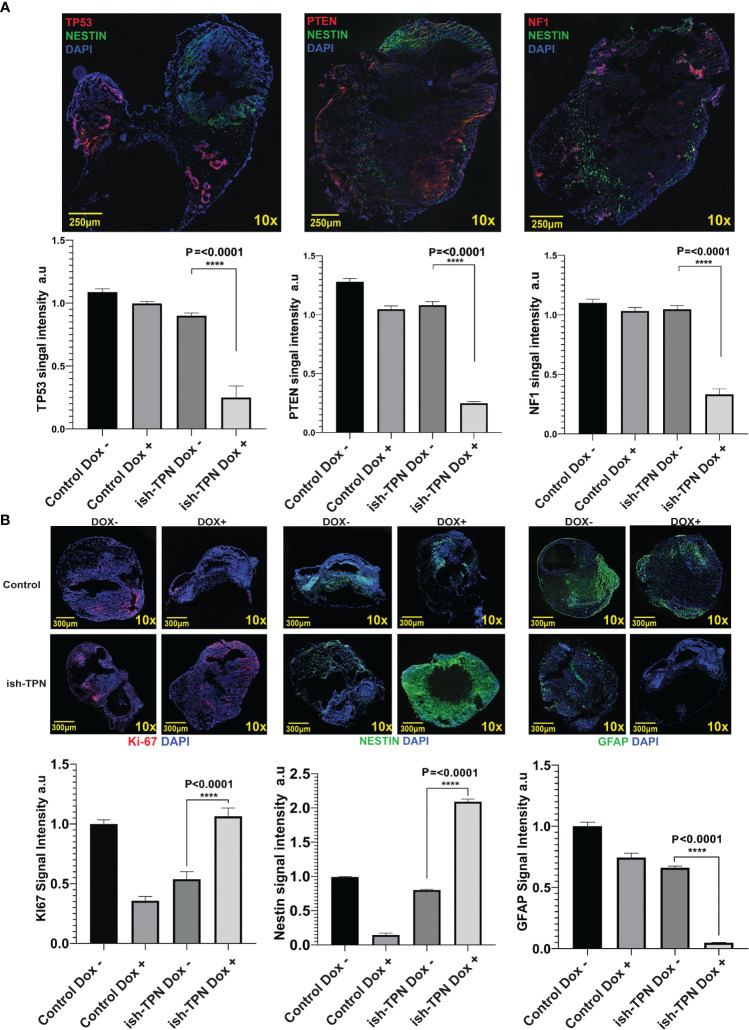
Organoid cancer model characterization **(A)** Representative Immunofluorescence images of ish-TPN doxycycline-induced cancer organoids (top panel): Neural stem cell marker Nestin (green), tumor suppressor markers TP53 (left), PTEN (middle), or NF1 (right) (each in red), with DAPI-stained nuclei (blue). Quantification of the signal intensity for the three targeted proteins (TP53, PTEN, and NF1) in Nestin-positive areas in the control (no ish-TPN) cerebral organoids (n=8) and ish-TPN containing cancer organoids (n=7) with and without doxycycline treatment (lower panel). **(B)** Immunofluorescence images of both control and ish-TPN-containing cancer organoids in Dox- and Dox+ conditions after labeling for Ki-67(red), Nestin, and GFAP (green) (top panel). Quantification of the signal intensity for Ki-67, Nestin, and GFAP (lower panel). Data values were represented in graphs as mean ± SEM. Images at 10x magnification were taken with a Nikon Eclipse Ti2-E microscope and analyzed with NIS-Elements AR, ver. 4.5. Magnification given in the caption reflect size before reduction. ****, highly significant.

Human GBM-like organoid sections were characterized for protein expression using immunofluorescence. Analysis for Nestin, TP53, PTEN, and NF1 revealed a significant (p <.0001) decrease of all 3 proteins only in the Nestin-positive areas (**Figure 5A**, top and **Figure 5A**, bottom). In fact, TP53, PTEN, and NF1 were markedly expressed only in areas where Nestin was absent, as seen in **Figure 5A**. This indicated a functional and sustained gene knockdown in the Nestin-positive niche. We quantitated these observations as detailed in the Methods section. Grid segmentation (**Supplementary Figure 7A**) of 60 organoids where regions of interest were identified revealed that all three gene products (TP53, PTEN, and NF1) were significantly knocked down in COs containing ish-TPN Dox+ compared to the other three groups (p <.0001). This was consistent with our immunofluorescence images where the loss of TP53, PTEN, and NF1 was apparent and did not appear in areas where Nestin was positive (**Supplementary Figure 7A**). PTEN showed the most knockdowns, followed by TP53, with NF1 showing the least knockdown (**Figure 5A**). We then investigated proliferation predilections within the 4 test groups using Ki-67 quantification. We implemented the same acquisition and analysis methods as described above. Ki-67 signal quantification showed a significant increase in the ish-TPN Dox+ COs compared to the ish-TPN Dox- COs, but no significant difference when compared to the control Dox- (no shRNAs) test group (**Figure 5B**). Nestin quantification was also investigated among the 4 test groups. Since Nestin is the promoter of our proposed construct, we anticipated Nestin being mostly expressed within the ish-TPN Dox+ group. Signal quantification showed a significant increase of Nestin signal from the ish-TPN Dox+ compared to other test groups (p <.0001) (**Figure 5B**). GFAP signal recorded the lowest intensity within the ish-TPN Dox- test group, and the highest within the control Dox- group (**Figure 5B**, right panel).

## 4 Discussion

Currently, several 3D GBM cultures have been described, owing to their value in addressing normal and disease brain development, including a faithful representation of the histoarchitecture. The production of these organoids ranges from a spheroid culture of patient-derived cancer cells, incorporation of glioma stem cells from patients into pre-formed organoids, and electroporation, to constitutively overexpressed genes within the organoid and production of organoids for potential personalized medicine development, to cutting resected patient tumors and generating sphere-like organoids or ex-vivo assembloids/slice cultures (15–17, 21, 32, 42, 43). However, there are no inducible and defined niche-specific genetically engineered human cancer organoids described in the literature to our knowledge, We have taken the approach of creating a doxycycline-inducible triple-targeting (TP53-PTEN-NF1) shRNA method and compared it to un-induced human cerebral organoids.

A key feature of our method is an inducible and genetically defined brain cancer organoid model that permits the direct observation of human tumor initiation. We achieved a roughly 40% knockdown of TP53 and a 50% knockdown of PTEN and NF1. Genetical-ly-engineered models have been successful in developing GBM-like tumors (15, 17). Nevertheless, here in our ish-TPN genetically-engineered brain organoid, we have shown that it displays key features of cancer, including somatic mutations, histopathology reminiscent of GBM, cellular identities, and copy number aberrations consistent with a proneural GBM subtype signature. Gene set enrichment analysis performed on the dif-ferentially-expressed genes revealed that genes that have been associated with GBM were found in our human GBM-like organoids, such as BCAN, a GBM-invasive marker, KPNA2, which is associated with metabolic reprogramming, and, notably, upregulatedSOX4, consistent with what others have described (32, 44, 45). Some of the most notable dif-ferentially-expressed genes, however, included GATA4, a tumor suppressor that was downregulated and is a negative regulator of survival and a prognostic marker (46). Interestingly, we also identified CBR1 downregulation, which catalyzes the reduction of the antitumor anthracyclines doxorubicin and daunorubicin and is one of the most downregulated genes in the proneural GBM subtype (47). This could be our potential treatment target for future studies.

We believe that future modifications or manipulations of oncogenes and/or tumor suppressor genes will allow for the development of all GBM subtypes in a human GBM-like CO model. In our case, ish-TPN under the control of a Nestin promoter produced sites of induced abnormal growth within the SVZ-like region of human COs, a region that is home to stem cells purported to be the site of GBM formation (6, 22, 48). Although the targeting of these tumor suppressor genes has been accomplished in mouse models, our ish-TPN COs work on a much shorter time frame to develop tumors-the addition of Dox to our human COs containing our targeted tumor suppressor genes forms GBM-like tumors in 2 weeks’ time. This model eliminates host-tumor discrepancies with mouse models that develop tumors by the incorporation of human cells into a mouse or a primarily mouse-tumor-derived GBM. Our model, similar to others, has a lack of functional vasculature and is therefore limited in its ability to explore neovascularization brought about by GBM formation. Our current approach for deriving GBM-like ish-TPN from hESCs can be conveniently applied in human inducible pluripotent stem cells (hIPSCs) as described by others (13, 14, 16). which offers an opportunity to study patient-specific tumors, GBM heterogeneity, and personalized medicine.

## 5 Conclusions

Our findings demonstrated a GBM-like phenotype and genotype within organoids with triple knockdown of TP53, NF1, and PTEN. To our knowledge, no organoid triple gene knockdown has been described as the reported ish-TNP here. The phenotype represents a significant step towards a more faithful glioma model by pathological analysis. Additionally, through the inferred mutational landscape analysis of our RNA sequencing data and comparison to TCGA glioblastoma library, we found the ish-TNP COs had a proneural expression signature of GBM. Our work-in-progress is to target key genes to generate a personalized glioma-like organoid system that to a great extent could recapitulate each patient’s individualized glioma landscape and enable clinicians to employ the Glioma-like organoid as a bio-factory to test and train therapeutic agents (e.g., oncolytic viruses, tumor-infiltrating lymphocytes, etc.). we believe in the near future, human Glioma-like organoid models could provide a realistic option in moving towards a personalized patient-less glioma therapy clinical trials paradigm.

## Data availability statement

The datasets presented in this study can be found in online repositories. The names of the repository/repositories and accession number(s) can be found in the article/**Supplementary Material**.

## Ethics statement

Ethical approval was not required for the studies on humans in accordance with the local legislation and institutional requirements because only commercially available established cell lines were used. Ethical approval was not required for the studies on animals in accordance with the local legislation and institutional requirements because only commercially available established cell lines were used.

## Author contributions

SS: Conceptualization, Data curation, Investigation, Methodology, Writing – original draft. YW: Data curation, Formal Analysis, Investigation, Project administration, Software, Writing – original draft. AH: Data curation, Formal Analysis, Investigation, Methodology, Software, Visualization, Writing – original draft, Writing – review & editing. MP: Data curation, Formal Analysis, Investigation, Software, Writing – original draft. CK: Formal Analysis, Investigation, Methodology, Software, Supervision, Writing – original draft, Writing – review & editing. AC: Data curation, Formal Analysis, Methodology, Project administration, Validation, Writing – original draft. WM: Data curation, Formal Analysis, Investigation, Methodology, Software, Supervision, Writing – original draft. JW: Formal Analysis, Methodology, Writing – original draft. NH: Project administration, Writing – original draft. BH: Data curation, Methodology, Writing – review & editing. GF: Data curation, Methodology, Supervision, Writing – review & editing. SK: Data curation, Formal Analysis, Investigation, Writing – review & editing. NA: Methodology, Validation, Writing – review & editing. RC: Methodology, Supervision, Validation, Writing – review & editing. LE: Data curation, Investigation, Methodology, Writing – review & editing. PZ: Conceptualization, Data curation, Investigation, Project administration, Validation, Writing – review & editing.

## Supplementary material

Supplementary Figure 1 Verifying Cerebral Organoid Markers and shRNA Targeted K¬¬¬nockdown, related to **Figure 1**. **(A)** Western blot for key pluripotency factors in hESCs demonstrates their stem cell state during cerebral organoid development. **(B, C)**, Western blots demonstrating knockdown of TP53, PTEN, and NF1 in neural stem cells after infection with shRNA constructs targeting TP53, PTEN, and NF1.

Supplementary Figure 2 Neural Stem Cell Characteristics Upon Doxycycline Induction, related to **Figure 2**. **(A)** Real-time PCR indicating decreased Ki-67 expression under the conditions indicated in NSCs **(B)** Real-time PCR indicating Nestin expression under the conditions indicated in NSCs. *p indicates <0.05.

Supplementary Figure 3 The landscape of Human GBM-like Cerebral Organoids, related to **Figure 4**. **(A)** Integrated analysis of human GBM COs depicting amplifications (red), deletions (blue), and mutations (green) encompassing missense mutations, splice-site mutations, and insertions. **(B)** Contribution of the mutation type shown in **(A)** to the point mutation spectrum in human GBM-like COs. **(C)** The relative contribution of the 96 trinucleotide changes to the original mutational spectrum (top panel) and the reconstructed mutational profile (middle panel), and the difference between these profiles (lower panel).

Supplementary Figure 4 Determining the Inter- and Intra-group Variability, related to **Figure 4**. **(A)** Principal component analysis (PCA) displaying 17 samples along the PC2, PC1, and PC3 axis accounting for 62%, 20%, 18% of the variability, respectively, within the gene expression set consisting of Group1: ish-TPN Dox+ cerebral organoids (i.e., induced to form human GBM-like COs), Group 2: cerebral organoids with no ish-TPN cassette- doxycycline-treated, and Group 3: ish-TPN Dox- cerebral organoids. **(B)** A plot of Pearson’s correlation between samples.

Supplementary Figure 5 Classification of Human GBM COs, related to **Figure 4**. **(A)** Heatmap visualizing unsupervised hierarchical clustering of genes expressed between Dox- and Dox+ ish-TPN cancer organoids and control organoids with no ish-TPN construct but treated with Dox. **(B)** Volcano plot showing differentially expressed genes in human GBM-like COs treated with Dox, with upregulated genes (red) and downregulated genes (blue), with the magnitude of fold changes shown on the x-axis and the statistical significance shown on the y-axis. Genes with a fold change of less than 2 are shown in gray. **(C)** TCGA sample data set shows a proper prediction of GBM subtypes: proneural, classical, neural, and mesenchymal. **(D)** The cerebral organoid dataset using the same algorithm under the same condition indicates that the GBM-like cerebral organoids are of the proneural subtype. **(E)** Gene set enrichment analysis of the GBM-like cerebral organoids also indicates the proneural GBM subtype.

Supplementary Figure 6 Gene and Disease Ontology of Human GBM COs, related to **Figure 4**. **(A)** Gene ontology enrichments are shown for differentially expressed genes. **(B)** Disease ontology enrichments are shown for differentially expressed genes.

Supplementary Figure 7 Representative Image Analysis of the Cancer Organoid, related to **Figure 5**. **(A)** Immunofluorescence image analysis of the entire stitched organoid section overlaid with a non-overlapping grid. 100 squares were represented in each grid, any square not covering the organoid area was trimmed. Signal intensity was measured in each square across all the channels. **(B)** Immunofluorescence images representing the cancer organoid in which sections were stained with antibodies against Nestin (green, left column) TP53, PTEN, or NF1 (red, middle column) and merged images of both channels and DAPI (Nuclei-blue) right column. Images were taken in 10x magnification.

Supplementary Figure 8 Representative images showing the proliferative index difference as organoids mature mimic the infant and adult human brain.
