# Supplementary figures and images for: TP53-PTEN-NF1 depletion in human brain organoids produces a glioma phenotype in vitro
Source: Front Oncol. 2023 Oct 10;13:1279806. doi: 10.3389/fonc.2023.1279806 (PMC10597663; doi:10.3389/fonc.2023.1279806)

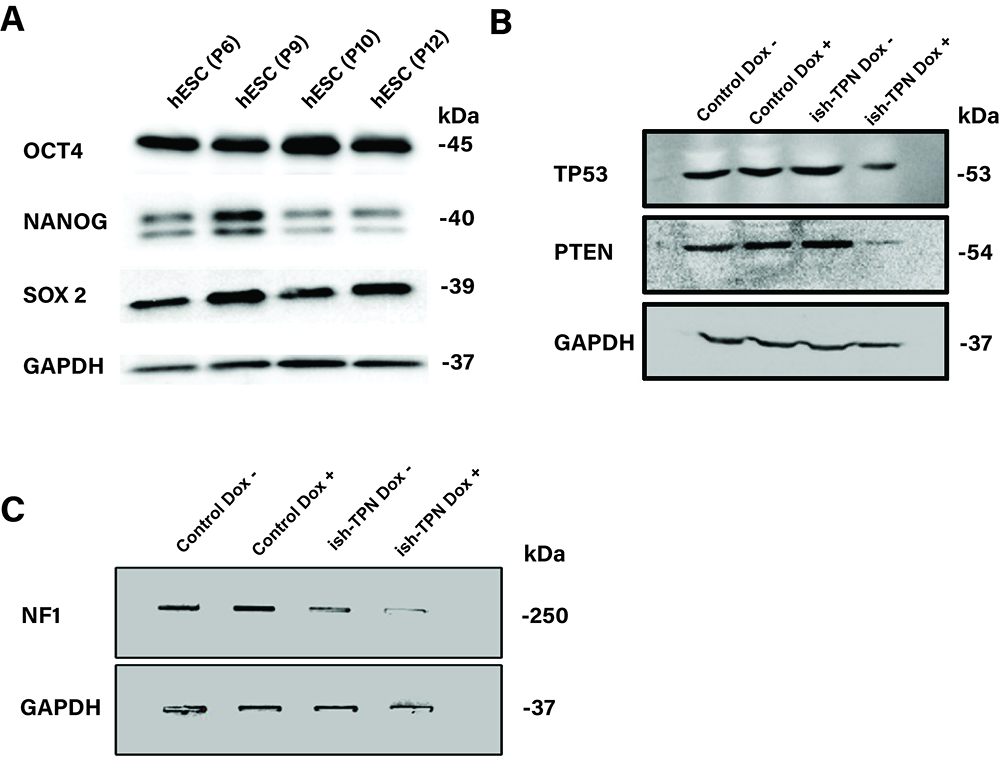

Supplement: Supplementary Figure 1 — Verifying Cerebral Organoid Markers and shRNA Targeted K¬¬¬nockdown, related to Figure 1 . (A) Western blot for key pluripotency factors in hESCs demonstrates their stem cell state during cerebral organoid development. (B, C), Western blots demonstrating knockdown of TP53, PTEN, and NF1 in neural stem cells after infection with shRNA constructs targeting TP53, PTEN, and NF1. [file DataSheet_1.zip › Habib - Organoid Paper FIG-1 - Supplemental .tif]

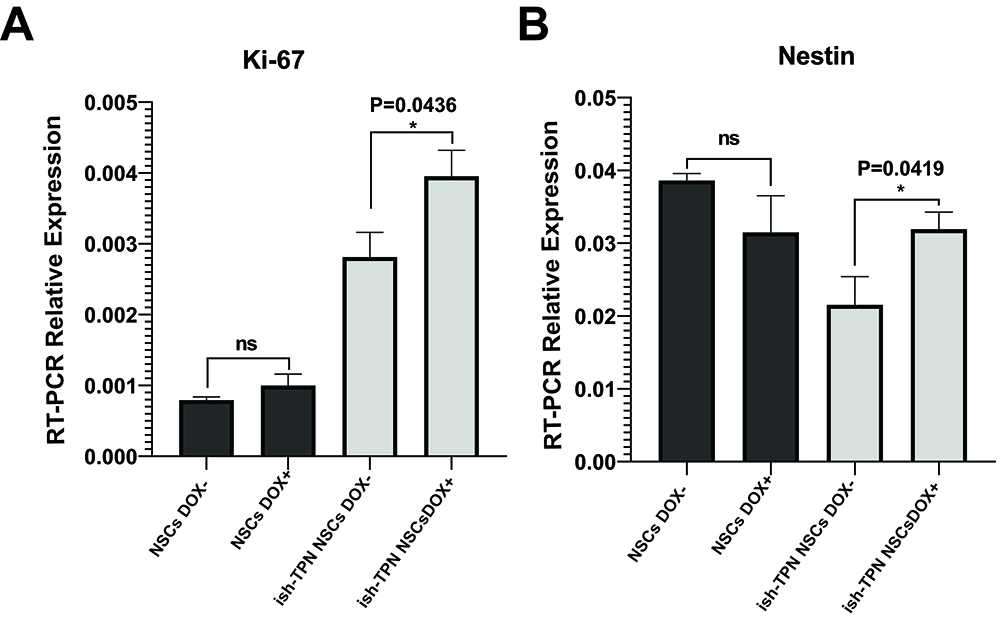

Supplement: Supplementary Figure 1 — Verifying Cerebral Organoid Markers and shRNA Targeted K¬¬¬nockdown, related to Figure 1 . (A) Western blot for key pluripotency factors in hESCs demonstrates their stem cell state during cerebral organoid development. (B, C), Western blots demonstrating knockdown of TP53, PTEN, and NF1 in neural stem cells after infection with shRNA constructs targeting TP53, PTEN, and NF1. [file DataSheet_1.zip › Habib - Organoid Paper FIG-2 - Supplemental .tif]

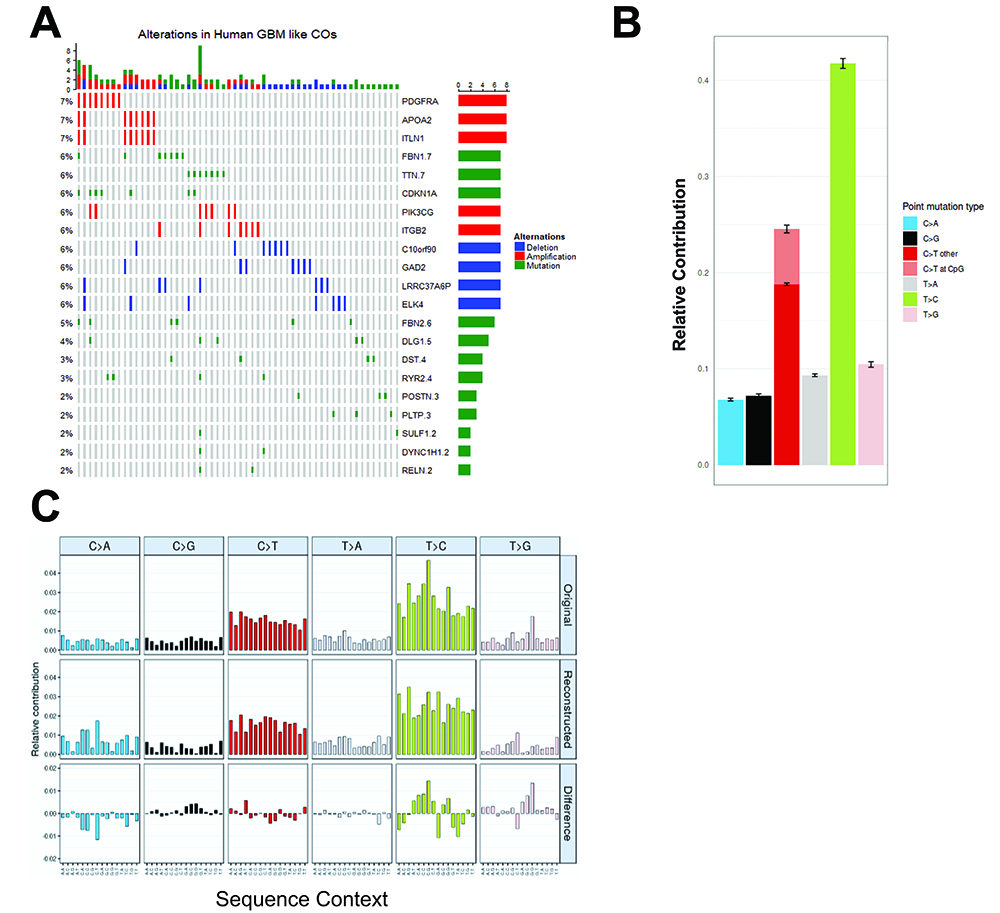

Supplement: Supplementary Figure 1 — Verifying Cerebral Organoid Markers and shRNA Targeted K¬¬¬nockdown, related to Figure 1 . (A) Western blot for key pluripotency factors in hESCs demonstrates their stem cell state during cerebral organoid development. (B, C), Western blots demonstrating knockdown of TP53, PTEN, and NF1 in neural stem cells after infection with shRNA constructs targeting TP53, PTEN, and NF1. [file DataSheet_1.zip › Habib - Organoid Paper FIG-3 - Supplemental .tif]

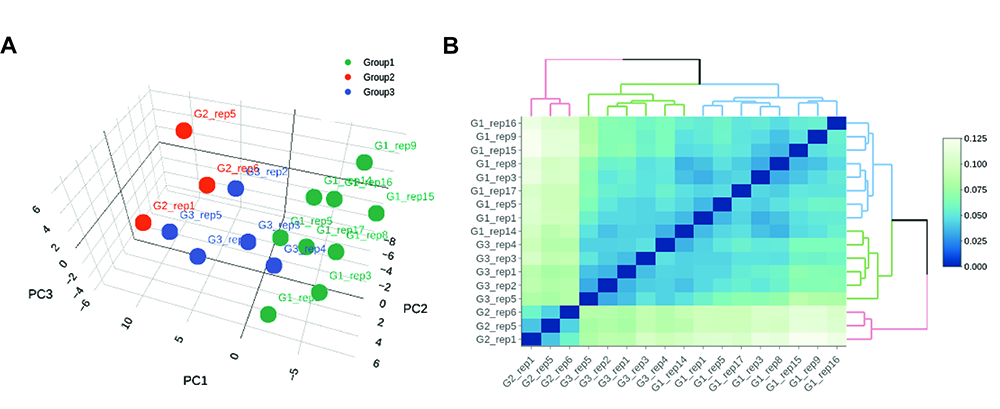

Supplement: Supplementary Figure 1 — Verifying Cerebral Organoid Markers and shRNA Targeted K¬¬¬nockdown, related to Figure 1 . (A) Western blot for key pluripotency factors in hESCs demonstrates their stem cell state during cerebral organoid development. (B, C), Western blots demonstrating knockdown of TP53, PTEN, and NF1 in neural stem cells after infection with shRNA constructs targeting TP53, PTEN, and NF1. [file DataSheet_1.zip › Habib - Organoid Paper FIG-4 - Supplemental .tif]

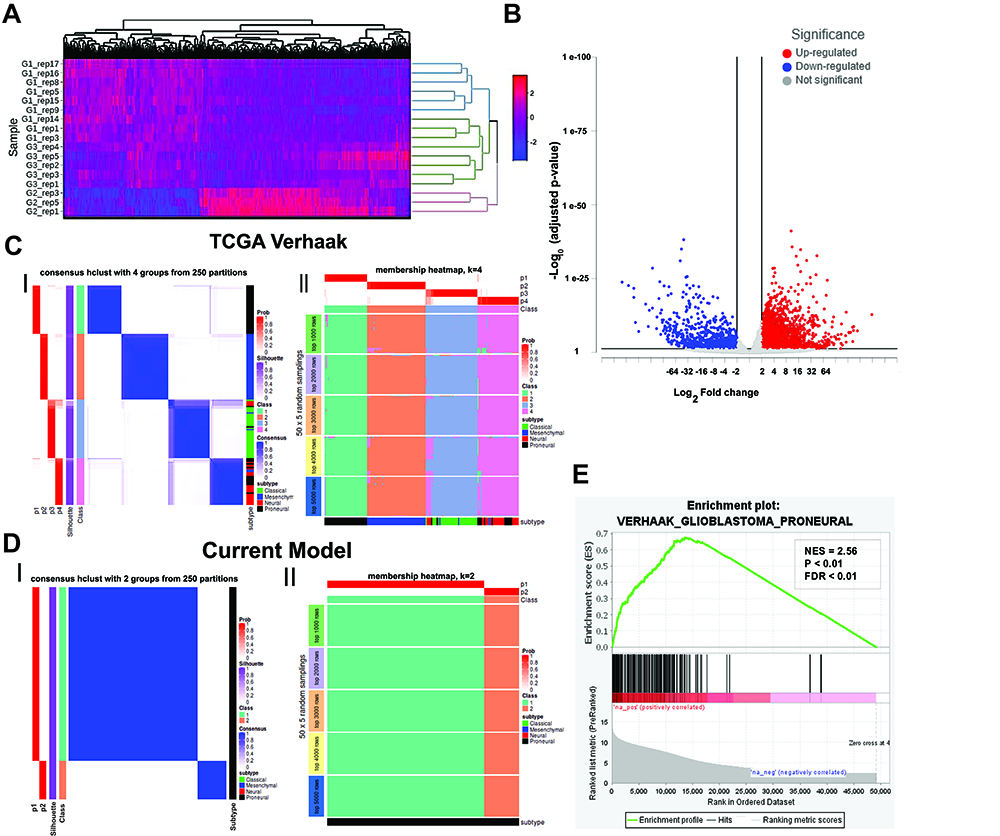

Supplement: Supplementary Figure 1 — Verifying Cerebral Organoid Markers and shRNA Targeted K¬¬¬nockdown, related to Figure 1 . (A) Western blot for key pluripotency factors in hESCs demonstrates their stem cell state during cerebral organoid development. (B, C), Western blots demonstrating knockdown of TP53, PTEN, and NF1 in neural stem cells after infection with shRNA constructs targeting TP53, PTEN, and NF1. [file DataSheet_1.zip › Habib - Organoid Paper FIG-5 - Supplemental .tif]

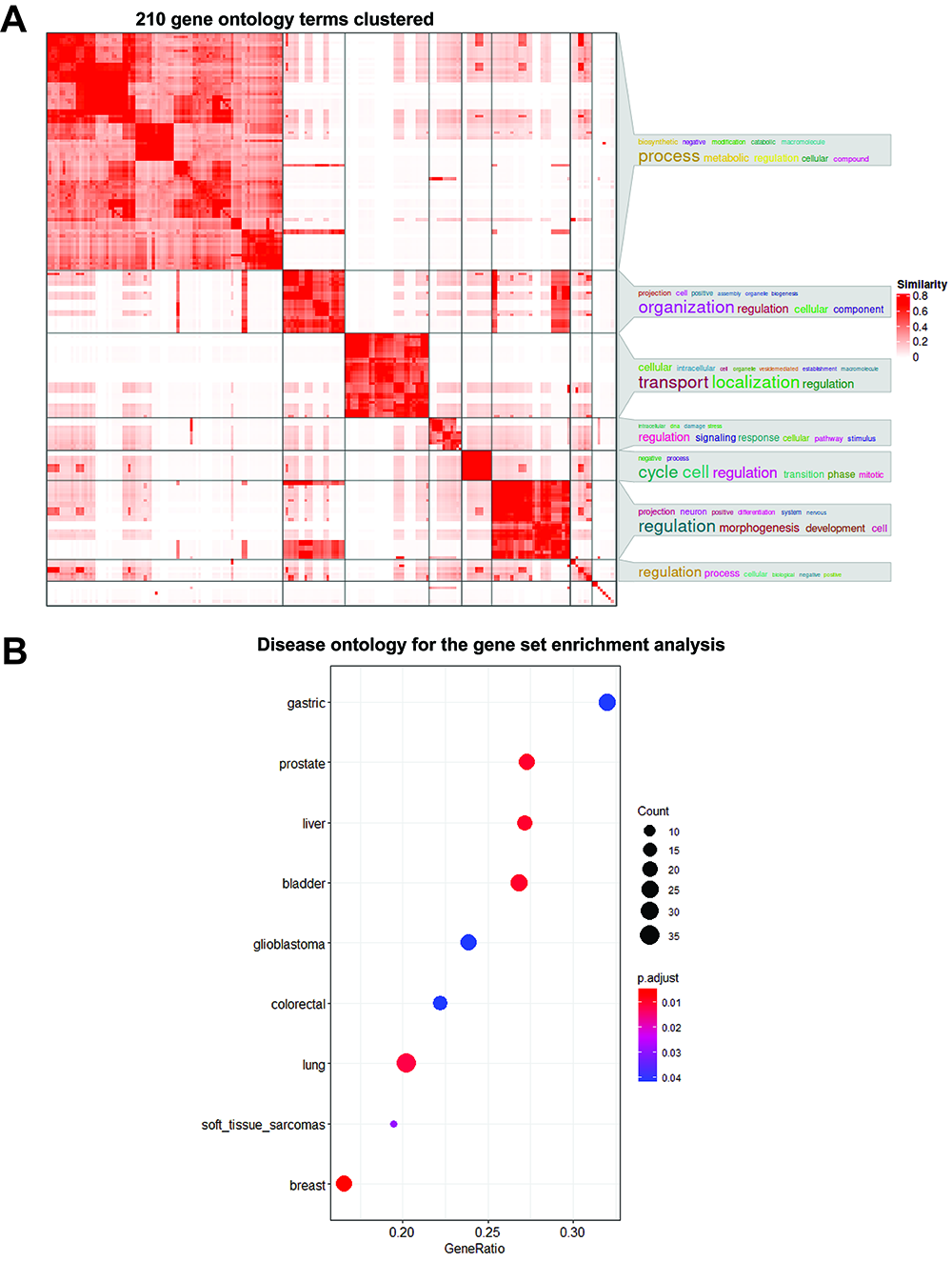

Supplement: Supplementary Figure 1 — Verifying Cerebral Organoid Markers and shRNA Targeted K¬¬¬nockdown, related to Figure 1 . (A) Western blot for key pluripotency factors in hESCs demonstrates their stem cell state during cerebral organoid development. (B, C), Western blots demonstrating knockdown of TP53, PTEN, and NF1 in neural stem cells after infection with shRNA constructs targeting TP53, PTEN, and NF1. [file DataSheet_1.zip › Habib - Organoid Paper FIG-6 - Supplemental .tif]

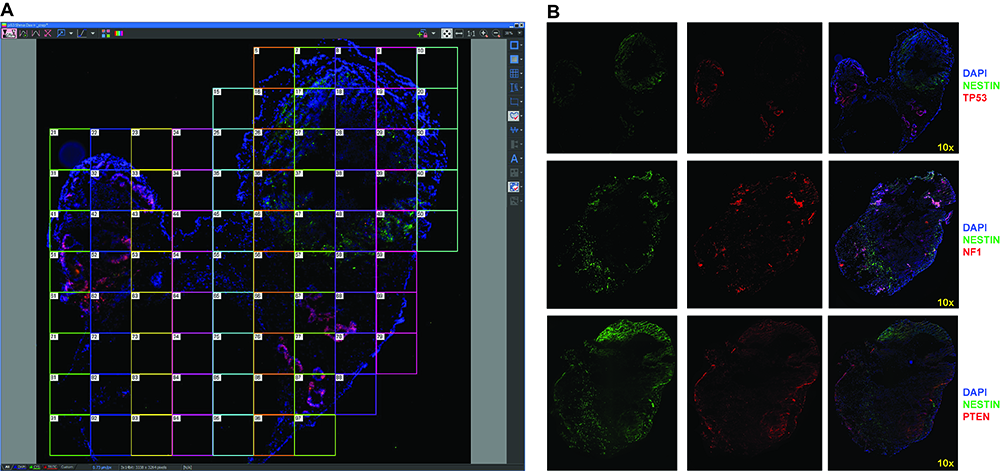

Supplement: Supplementary Figure 1 — Verifying Cerebral Organoid Markers and shRNA Targeted K¬¬¬nockdown, related to Figure 1 . (A) Western blot for key pluripotency factors in hESCs demonstrates their stem cell state during cerebral organoid development. (B, C), Western blots demonstrating knockdown of TP53, PTEN, and NF1 in neural stem cells after infection with shRNA constructs targeting TP53, PTEN, and NF1. [file DataSheet_1.zip › Habib - Organoid Paper FIG-7 - Supplemental .tif]

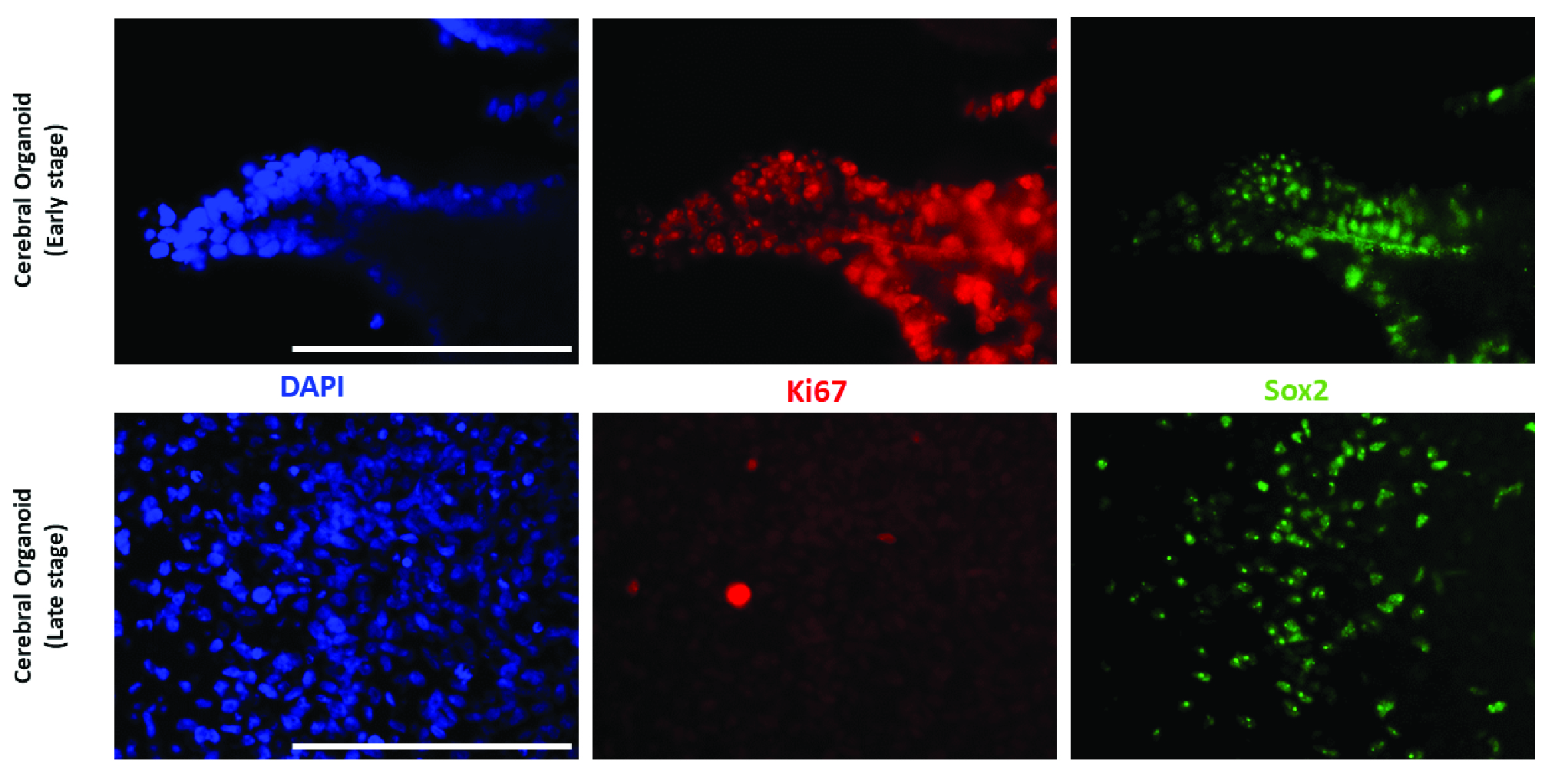

Supplement: Supplementary Figure 1 — Verifying Cerebral Organoid Markers and shRNA Targeted K¬¬¬nockdown, related to Figure 1 . (A) Western blot for key pluripotency factors in hESCs demonstrates their stem cell state during cerebral organoid development. (B, C), Western blots demonstrating knockdown of TP53, PTEN, and NF1 in neural stem cells after infection with shRNA constructs targeting TP53, PTEN, and NF1. [file DataSheet_1.zip › Habib - Organoid Paper FIG-8 - Supplemental - 06_30_2021.jpg]
