# Supplementary material for: TP53-PTEN-NF1 depletion in human brain organoids produces a glioma phenotype in vitro
Source: Front Oncol. 2023 Oct 10;13:1279806. doi: 10.3389/fonc.2023.1279806 (PMC10597663; doi:10.3389/fonc.2023.1279806)

**SUPPLEMENTARY TABLES:**

**Table S1**. shRNA oligo sequences used to construct vectors.


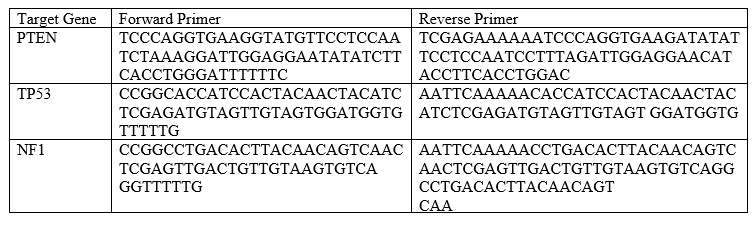


**Table S2.** qRT-PCR primers used in the study.


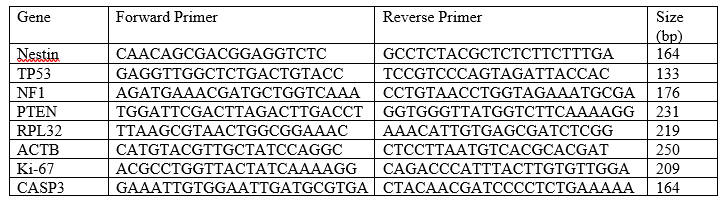

Supplement: Supplementary file 2 [file Table_1.docx]
